# Dosimetric analysis of orthogonal collimator configuration in volumetric modulated arc therapy planning: a comparative study

**DOI:** 10.3389/fonc.2025.1612643

**Published:** 2025-07-29

**Authors:** Xin Huang, Chenlei Guo, Shuangtong Liu, Kuo Men, Hui Wang

**Affiliations:** ^1^ Department of Oncology, Tianjin Union Medical Center, The First Affiliated Hospital of Nankai University, Tianjin, China; ^2^ Tianjin Cancer Institute of Integrative Traditional Chinese and Western Medicine, Tianjin Union Medical Center, The First Affiliated Hospital of Nankai University, Tianjin, China; ^3^ Department of Radiation Oncology, National Cancer Center/National Clinical Research Center/Cancer Hospital, Chinese Academy of Medical Sciences and Peking Union Medical College, Beijing, China

**Keywords:** radiotherapy, radiotherapy treatment planning, volumetric modulated arc therapy, orthogonal collimator angles, dose conformity and homogeneity

## Abstract

**Background:**

In volumetric modulated arc therapy (VMAT), collimator angle selection impacts dose distribution and plan quality. Conventional VMAT plans use dual arcs with collimators set at 0˚. This study explores the dosimetric effects of using orthogonal collimator angles (0˚ and 90˚) in dual-arc VMAT.

**Methods:**

Sixty patients with head and neck, thoracic, and abdominal tumors were analyzed. Two VMAT plans were generated: Plan A (0˚ collimator angle) and Plan B (0˚ and 90˚ collimator angles). Dosimetric endpoints included conformity index (CI), gradient measure (GM), homogeneity index (HI), dose to organs at risk (OARs), mean dose to normal tissues (D_mean, NT_), monitor units (MU), and gamma pass rate (GPR).

**Results:**

Plan B improved dosimetric outcomes over Plan A. HI decreased by 0.03 in the rectum, 0.01 in the breast, and 0.01 in the larynx. GM decreased by 0.15 cm in the rectum, 0.05 cm in the breast, and 0.01 cm in the larynx. OAR doses were reduced across sites, with notable decreases in the bladder (−4.62 Gy), left anterior descending artery (−3.99 Gy), and spinal cord (−1.79 Gy). D_mean,NT_ was slightly reduced in the rectum, breast and larynx. MU increased in rectum plans (+41 MU), but decreased in the breast and laryngeal plans by 38 MU and approximately 73 MU, respectively. All plans achieved GPR > 95%.

**Conclusion:**

Incorporating orthogonal collimator angles (0˚ and 90˚) in dual-arc VMAT enhances dose conformity and spares OARs without compromising target coverage or delivery accuracy. This approach is clinically applicable with minimal workflow changes.

## 1 Background

In the field of radiation therapy, volumetric modulated arc therapy (VMAT) achieves high-precision irradiation of planning target volume through continuous gantry rotation and dynamic adjustment of the multileaf collimator (MLC) while maximizing the protection of the surrounding normal tissues (1–3). The selection of collimator angles has a significant effect on the dose distribution and plan quality of VMAT (4–6). Currently, patients undergoing radiation therapy typically receive double-arc VMAT plans with a collimator angle of 0˚ (5, 7–10).

Under the conventional double-arc field with a collimator angle of 0˚, the beam primarily irradiates the planning target along the anteroposterior direction (i.e., from the front to the back of the patient or vice versa) (11). This arrangement may lead to several limitations. The movement direction of the MLC leaves is parallel to the gantry rotation plane, restricting the flexibility of the leaves in the direction perpendicular to the gantry rotation (3, 12, 13). Additionally, the beam shape of the 0˚ collimator is relatively fixed and may not adapt well to complex planning target shapes and the protection needs of the surrounding normal tissues (7, 14, 15). In particular, when the planning target has a pronounced concave shape or a large curvature, the 0˚ collimator may fail to effectively avoid critical organs, leading to increased dose exposure and potential radiation-induced side effects (16). For organs with relatively flexible positioning, such as the small intestine in the pelvic cavity, the 0˚ collimator arrangement may lead to high doses in certain regions, increasing the risk of small intestine damage such as enteritis or intestinal obstruction (17, 18).

To reduce the dose exposure of normal tissues, this study introduced a 90˚ collimator angle into a conventional double-arc VMAT plan. This setting leverages the modulating capability of the MLC in the orthogonal direction, which is expected to improve the dose distribution in the planning target and protect critical normal organs. This study aimed to explore a simple and effective optimization method to enhance the quality and therapeutic effects of VMAT plans without significantly increasing the plan complexity. These findings aim to provide a new perspective for the design of radiation therapy plans, particularly in improving treatment efficiency and protecting normal tissues.

## 2 Methods

### 2.1 Case selection

This retrospective study randomly selected 60 patients who underwent VMAT at the Tianjin People’s Hospital in December 2024. The patient cohort included 20 patients each with laryngeal cancer, 20 with left-sided breast cancer, and 20 with rectal cancer. The prescribed doses for the target volumes were set at 60.06 Gy/33 fractions for laryngeal cancer, 50 Gy/25 fractions for breast cancer, and 48.6 Gy/27 fractions for rectal cancer. The study adhered to the guidelines of the medical ethics committee, and all patient data were anonymized and analyzed. All patients underwent computed tomography (CT) simulation using a 16-slice large-bore CT scanner (General Electric Company. (2015). Discovery RT 590. GE Healthcare).

### 2.2 Plan design

For each patient, the planning target volume (PTV) and organs at risk (OARs) were contoured using Eclipse Treatment Planning System based on patient imaging data. Two VMAT plans were designed and labeled Plan A and Plan B. Plan A used a conventional double-arc plan with a collimator angle of 0˚, whereas Plan B employed a double-arc plan with orthogonal collimator angles of 0˚ and 90˚. All other parameters, such as the beam energy, field size, and optimization objectives, were kept consistent during the planning process, with only the collimator angles being altered. All plans were designed and optimized by the same medical physicist to ensure the reliability and accuracy of the results. In this study, all treatment plans for patients with rectal cancer were standardized to ensure that 100% of the target volume received at least 95% of the prescribed dose.

### 2.3 Target evaluation

The dose distributions of the target volumes for both plans were meticulously assessed using the conformity index (CI) (19, 20), gradient measure (GM) (21), and homogeneity index (HI) (22).

The CI is a crucial quantitative metric in radiation therapy used to evaluate the conformity of the high-dose region to the PTV. A CI value closer to 1 indicated better conformity between the dose distribution and target, indicating that the shape and size of the high-dose region more closely matched the target. CI was calculated using the following formula:


$$CI=\frac{V_{PTV,ref}}{V_{PTV}}\times \frac{V_{ref}}{V_{PTV}}$$

where, $V_{PTV,ref}$ is the target volume at the prescribed dose, $V_{PTV}$ is the PTV volume, and $V_{ref}$ is the total volume covered by the prescribed dose.

The GM assesses the dose fall-off at the target margins, serving as an important parameter for evaluating the dose transition between the target and surrounding normal tissues. The GM is typically measured in centimeters, representing the distance from the target edge to the 50% prescription isodose line. A higher GM value indicates faster dose fall-off at the target edge, resulting in better protection of the surrounding normal tissues. GM was calculated as follows:


$$GM=\sqrt[{3}]{\frac{3V_{50\%}}{4\pi }}-\sqrt[{3}]{\frac{3V_{PTV}}{4\pi }}$$

where V_50%_ is the volume enclosed by the 50% prescription isodose line, and $V_{PTV}$ is the PTV volume.

The HI is a vital metric for evaluating the uniformity of the dose distribution within a target during radiation therapy. An HI value closer to 0 indicates a more uniform dose distribution within the target. HI was calculated using the following formula:


$$HI=\frac{D_{2}-D_{98}}{D_{50}}$$

where D_2_ is the dose covering 2% of the target volume, D_98_ is the dose covering 98% of the target volume, and D_50_ is the dose covering 50% of the target volume, respectively.

### 2.4 OARs evaluation

In radiation therapy, dosimetric evaluation of OARs is crucial for ensuring treatment safety. The OARs of interest are listed in **Table 1**. The mean dose (D_mean, OAR_) to OARs was used to assess the overall dose exposure, whereas the maximum dose (D_max_) was used to evaluate potential high-dose risks. Additionally, the ratio of total body volume receiving a specific percentage of the prescribed dose (20%, 40%, 60%, and 80%) was assessed and denoted as V_20%_, V_40%_, V_60%_, and V_80%_, respectively.

**Table 1:** The mean and variance of evaluation indicators for Planning Target Volumes(PTV), Organs at Risk (OARs), Monitor units (MU) and the gamma pass rate in two plans for rectal cancer, breast cancer, and laryngeal cancer.

Disease	Target	Indicator	Plan A	Plan B	P value
Rectum	PTV	CI	1.10 ± 0.02	0.99 ± 0.01	0.023
		GM	3.06 ± 0.23	2.91 ± 0.20	0.002
		HI	0.11 ± 0.01	0.08 ± 0.00	<0.001
	Bladder	Mean dose	25.62 ± 5.63	21.00 ± 2.87	<0.001
	Pelvis	Mean dose	24.03 ± 3.28	22.92 ± 3.02	0.032
	Intestine	Mean dose	22.51 ± 3.70	20.87 ± 3.01	<0.001
		Max dose	49.54 ± 1.15	48.86 ± 0.84	0.002
	Femoral Left	Mean dose	19.33 ± 4.09	16.55 ± 2.69	0.001
	Femoral Right	Mean dose	20.52 ± 5.06	16.23 ± 3.34	0.001
	Body-PTV	D_mean,NT_	6.65 ± 0.90	6.53 ± 0.87	0.225
	Plan	MU	521 ± 100	562 ± 59	0.022
	QA	Gamma	97.14 ± 0.73	97.99 ± 0.38	<0.001
Breast	PTV	CI	1.01 ± 0.05	0.99 ± 0.04	0.015
		GM	1.68 ± 0.19	1.63 ± 0.17	0.150
		HI	0.13 ± 0.02	0.12 ± 0.02	0.003
	Breast	Mean dose	4.27 ± 1.83	4.08 ± 1.31	0.279
	Heart	Mean dose	6.44 ± 1.38	6.20 ± 1.30	0.271
	LAD	Mean dose	24.12 ± 4.87	20.13 ± 3.01	<0.001
	Lung Left	Mean dose	11.43 ± 1.84	10.83 ± 1.75	0.065
		V_5_	40.69 ± 10.24	42.61 ± 9.27	0.305
		V_20_	21.19 ± 6.97	18.52 ± 4.77	0.040
	Lung Right	Mean dose	3.80 ± 0.44	2.62 ± 0.54	0.211
	Body-PTV	D_mean,NT_	3.68 ± 0.45	3.49 ± 0.40	0.002
	Plan	MU	557 ± 70	519 ± 38	<0.001
	QA	Gamma	98.28 ± 0.77	98.58 ± 0.67	0.056
Larynx	PTV	CI	1.03 ± 0.03	1.03 ± 0.02	0.127
		GM	2.31 ± 0.36	2.30 ± 0.35	0.231
		HI	0.11 ± 0.01	0.10 ± 0.01	<0.001
	Mandible	Mean dose	28.71 ± 3.47	28.76 ± 3.66	0.723
		Max dose	61.16 ± 2.81	59.46 ± 1.32	0.002
	OC	Mean dose	30.77 ± 4.74	30.26 ± 4.935	0.029
	Parotid Left	Mean dose	21.52 ± 4.20	21.06 ± 4.12	0.588
	Parotid Right	Mean dose	22.25 ± 3.51	22.62 ± 3.14	0.737
	Pharyngeal	Mean dose	35.59 ± 7.67	33.05 ± 7.84	0.001
	Spinalcord	Max dose	19.77 ± 2.45	17.98 ± 1.90	<0.001
	Body-PTV	D_mean,NT_	8.55 ± 2.45	8.28 ± 2.59	0.162
	Plan	MU	587 ± 35	514 ± 8	<0.001
	QA	Gamma	96.98 ± 0.70	97.07 ± 0.62	0.808

The mean radiation dose to normal tissues (D_mean,NT_) refers to the average radiation dose from normal tissues outside PTV in the radiotherapy plan. This is an important indicator for assessing the safety of a radiotherapy plan and reflects the extent of radiation exposure to normal tissues. The equation for calculating D_mean,NT_ is as follows:


$$D_{mean,NT}=\frac{\left(V_{body}\times D_{mean,body})-(V_{PTV}\times D_{mean,PTV}\right)}{V_{body}-V_{PTV}}$$

where V_body_ is the total irradiated volume (e.g., body volume), D_mean,body_ is the mean dose to the total irradiated volume, V_PTV_ is the PTV volume, and D_mean,PTV_ is the mean dose to the PTV volume (with volume units in cm³ and dose units in Gy).

### 2.5 Plan evaluation

Monitor units (MU) are radiation therapy parameters that describe the total beam-on time required for a treatment plan. They reflect the irradiation intensity of the linear accelerator when executing the treatment plan. Under the same dose distribution, a lower total MU generally indicates a higher plan efficiency. In a treatment plan, the total MU is the sum of the MUs of all the beams.

Plan verification refers to the process of validating the dose distribution of a VMAT treatment plan during radiation therapy to ensure the accuracy and safety of the treatment plan. Since VMAT technology modulates volume by adjusting multiple parameters, such as the shape of the MLC opening, dose rate, and gantry rotation speed, its dose distribution is complex and requires individualized quality assurance (QA). In this study, an electronic portal imaging device (EPID) was used to compare the dose calculated using the planning system with the actual measured dose.

The gamma pass rate (GPR) is the percentage of points that meet specific dose difference and distance-to-agreement (DTA) criteria in gamma analysis. Gamma analysis was used to compare the planned dose distributions with measured dose distributions by calculating the gamma index for each point to assess the consistency between the two. Points with a gamma index < 1 are considered “passed” and the GPR is the percentage of these “passed” points out of the total evaluated points. In clinical practice, a commonly used gamma analysis criterion is a 3% dose difference and 3 mm DTA (3%/3 mm), with a pass rate of 90% being the acceptable standard. A higher GPR indicates better consistency between the planned and measured dose distributions, leading to higher treatment accuracy and safety.

### 2.6 Statistical analysis

A rigorous statistical treatment and analysis were conducted on the collected data. Comprehensive descriptive statistical analyses, including mean and standard deviation, were performed for all evaluation indicators encompassed by Plan A and Plan B. Given that the sample size for all analyses was only 20, the Kolmogorov–Smirnov (K–S) test (23) was employed to assess the normality of the data. The results of the normality test indicated that the majority of the data did not conform to a Gaussian distribution (p<0.05). Consequently, non-parametric methods were utilized for statistical comparisons. Specifically, the Wilcoxon signed-rank test (24) was applied for comparisons between the two groups. All statistical analyses were carried out using OriginPro 2024 software (OriginLab Corporation, Northampton, MA, USA). The significance threshold for this study was set at p<0.05.

## 3 Results

### 3.1 Target volume

**Figure 1:**
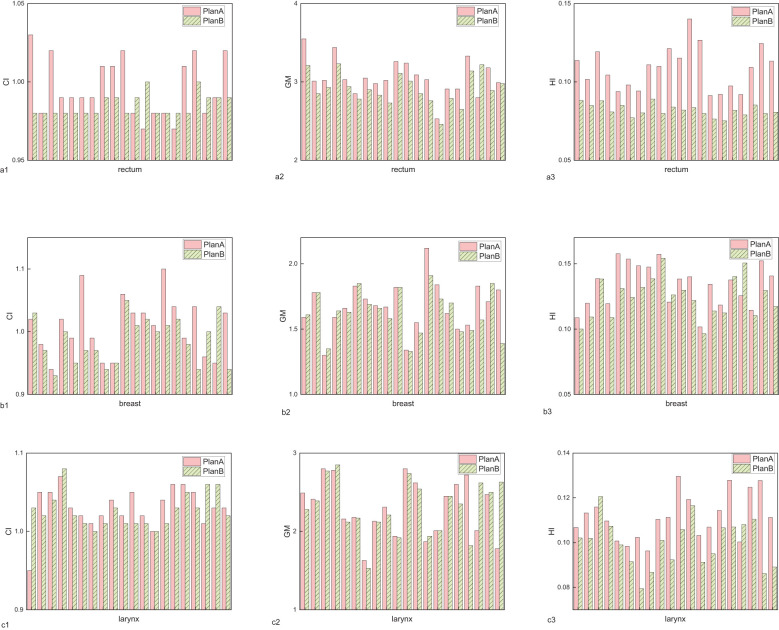
The assessment indicators for planning target volumes of rectal cancer, breast cancer, and laryngeal cancer. **(a)** conformity index (CI), **(b)** gradient measure (GM), **(c)** homogeneity index (HI).

In terms of target conformity, the CI for rectal, breast, and laryngeal cancers showed no significant differences between Plan A and Plan B, indicating consistent conformity between the two plans. The results can be seen in **Figure 1a** and **Table 1**. However, the average GM of Plan B was lower than that of Plan A across all three cancers, indicating a faster dose fall-off at the target margins and improved protection of surrounding normal tissues. Notably, the GM reduction in breast cancer was minimal (0.05 cm, p=0.150), and some individual cases exhibited higher GM in Plan B. The largest GM reduction was observed in rectal cancer (0.15 cm, p=0.002), followed by laryngeal cancer (0.01 cm). Notably, the reduction in breast cancer was minimal, suggesting a limited clinical impact and highlighting anatomical factors that may reduce the modulation advantage of orthogonal collimation in this site. The results are shown in **Figure 1b** and **Table 1**. Similarly, the HI of Plan B was lower than that of Plan A, indicating a more uniform dose distribution within the planning target. The HI reductions were 0.03 for rectal cancer (p<0.001), 0.01 for breast cancer (p=0.003), and 0.01 for laryngeal cancer (p<0.001). The results are presented in **Figure 1c** and **Table 1**. For clarity, P values are reported only when the comparison between plans resulted in notable or statistically significant differences.

### 3.2 OARs

**Figure 2:**
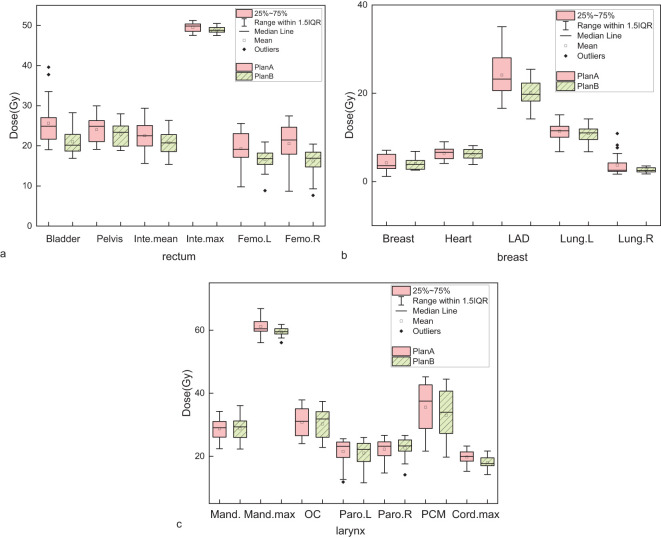
Comparison of mean or maximum dose to organs at risk (OARs) between Plan A (dual-arc VMAT with identical collimator angles) and Plan B (dual-arc VMAT with orthogonal collimator angles: 0˚ and 90˚). **(a)** Rectal cancer: bladder, pelvis, small intestine (Inte), left femoral head (Femo.L), and right femoral head (Femo.R); **(b)** Breast cancer: contralateral breast, heart, left anterior descending artery (LAD), ipsilateral lung (lung.L), and contralateral lung (lung.R); **(c)** Laryngeal cancer: mandible (Mand.), oral cavity (OC), left and right parotid glands (Paro.L and Paro.R), pharyngeal constrictor (PCM), and spinal cord. Each group contains 10 patients. Boxplots illustrate the distribution of dose values: boxes represent the interquartile range (IQR), horizontal lines indicate medians, whiskers extend to 1.5×IQR, and open circles denote statistical outliers. Slight dose reductions in Plan B are observed in several OARs, though absolute differences remain small in most cases.

For rectal cancer, Plan B demonstrated a reduction in the D_mean_ to the bladder, pelvis bone, intestine, and both femoral heads by 4.62 (p<0.001), 1.11 (p<0.001), 1.64 (p<0.001), left 2.78 (p=0.001) and right 4.29 Gy (p=0.001), respectively. The D_max_ to the intestine decreased by 0.68 Gy (p=0.002). D_mean,NT_ decreased from 6.65 ± 0.90 Gy in Plan A to 6.53 ± 0.87 Gy in Plan B. The results are presented in **Figure 2a** and **Table 1**.

For breast cancer, Plan B resulted in a decrease in the mean dose to the contralateral breast, heart, left anterior descending artery (LAD), ipsilateral lung and contralateral lung by 0.19, 0.24, 3.99 (p<0.001), 0.60, and 1.18 Gy, respectively. D_mean,NT_ decreased by 0.19 Gy (p=0.002). However, the volume of the affected lung receiving 5 Gy (V_5_:+1.92 Gy) increased, whereas the volume receiving 20 Gy (V_20_: -2.67 Gy) decreased. The results are shown in **Figure 2b** and **Table 1**.

For laryngeal cancer, the mean dose to mandible increased by 0.06 Gy, while the maximum dose decreased by 1.7 Gy (p=0.002). The mean doses to the oral cavity, left parotid gland, and pharyngeal constrictor decreased by 0.51 (p=0.029), 0.46, and 2.54 Gy (p=0.001), respectively. The mean dose to the right parotid gland increased by 0.37 Gy. The maximum dose to the spinal cord decreased by 1.79 Gy (p<0.001). D_mean,NT_ decreased by 0.27 Gy. The results can be seen in **Figure 2c** and **Table 1**.

### 3.3 Dose lines

**Figure 3:**
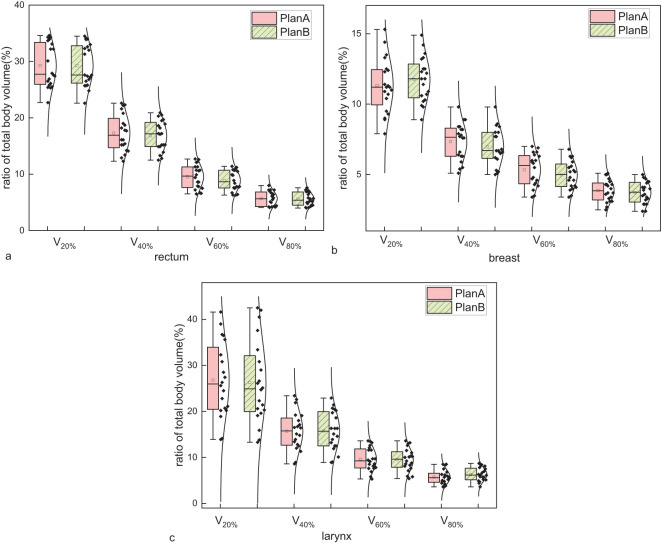
Comparison of the ratio of total body volume receiving 20%, 40%, 60%, and 80% of the prescription dose (V_20%_, V_40%_, V_60%_, and V_80%_) between Plan A and Plan B across three disease sites: **(a)** rectal cancer, **(b)** breast cancer, and **(c)** laryngeal cancer. Each metric reflects the percentage of the entire patient body exposed to respective isodose levels. Each group includes 20 patients. Boxplots represent distribution: the boxes show the interquartile range (IQR), horizontal lines indicate medians, whiskers extend to 1.5×IQR, and open circles represent outliers. Although Plan B showed numerically lower V-values in most cases, the differences were marginal, suggesting limited dosimetric impact on total body exposure.

In the analysis of isodose line volumes for rectal cancer, the ratios of total body volume receiving 20%, 40%, 60%, and 80% of the prescription dose (V_20%_, V_40%_, V_60%_, and V_80%_) were numerically lower in Plan B. However, these changes were small (typically <1%) and may reflect variability inherent to small-sample pilot data. The results are shown in **Figure 3a** and **Table 2**. For breast cancer, V_20%_ and increased by 0.43%. While V_40%_, V_60%_ and V_80%_ decreased by 0.37%, 0.36% and 0.15%, respectively. The results are displayed in **Figure 3b** and **Table 2**. For laryngeal cancer, V_40%_ and V_80%_ slightly increased by 0.03%, and 0.58%, respectively. While V_20%_ and V_60%_ decreased by 0.61%, and 0.09%, respectively. The results are displayed in **Figure 3c** and **Table 2**.

**Table 2:** Mean and variance of the ratio of total body volume corresponding to the prescribed dose percentages (20%, 40%, 60%, 80%).

Volume (cm^3^)		V_20%_	V_40%_	V_60%_	V_80%_
Rectum	Plan A	29.28 ± 3.87	17.29 ± 3.14	9.55 ± 2.13	5.67 ± 1.30
	Plan B	29.27 ± 3.77	16.80 ± 2.70	8.88 ± 1.82	5.59 ± 1.19
	Pvalue	0.295	0.161	0.001	0.671
Breast	Plan A	11.32 ± 1.91	7.35 ± 1.32	5.34 ± 1.16	3.88 ± 0.79
	Plan B	11.75 ± 1.60	6.98 ± 1.33	4.98 ± 1.00	3.73 ± 0.79
	Pvalue	0.108	0.711	0.004	0.016
Larynx	Plan A	26.84 ± 8.13	15.71 ± 4.21	9.61 ± 2.54	5.73 ± 1.56
	Plan B	26.23 ± 9.06	15.73 ± 4.27	9.52 ± 2.49	6.31 ± 1.47
	Pvalue	0.322	0.831	0.266	0.296

### 3.4 Dose-volume histogram

**Figure 4:**
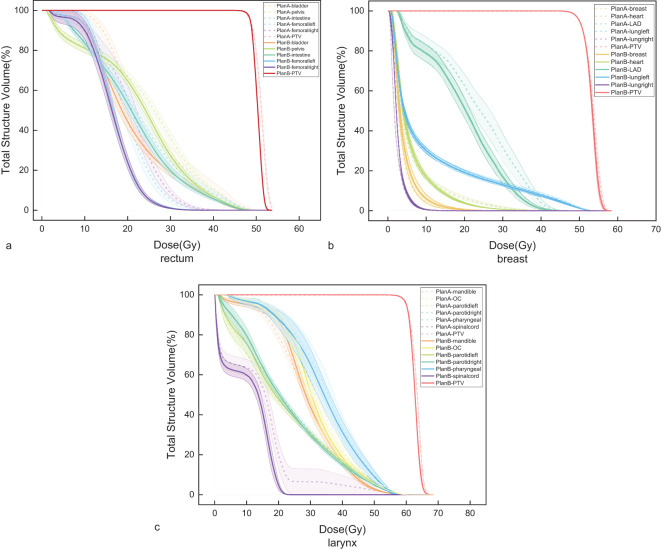
Dose-Volume Histogram (DVH) of the Planning Target Volumes(PTV) and Organs at Risk (OARs). **(a)** rectal cancer, **(b)** breast cancer, and **(c)** laryngeal cancer.

In the analysis of DVH for OARs, Plan B showed significant changes compared to Plan A. For rectal cancer, the DVH for the bladder, pelvis bone, and intestine showed a notable reduction. Specifically, the bladder DVH showed a maximum relative volume reduction of 19.57% within a dose range of 11.1–27.4 Gy. The pelvis bone DVH showed a maximum reduction of 9.23% within the dose range of 22.3–36.6 Gy. The intestine DVH showed a maximum reduction of 4.87% within the dose range of 11.2–40.4 Gy. The left femoral head DVH showed a maximum reduction of 21.51% within the dose range of 14.8–26.1 Gy, while the right femoral head DVH showed a maximum reduction of 31.86% within the dose range of 15.1–26.6 Gy. The results are shown in **Figure 4a**.

For breast cancer, Plan B showed an increase in the contralateral lung DVH within a dose range of 16.7–27.4 Gy, with a maximum increase of 0.15%. The contralateral breast DVH showed a maximum reduction of 0.15% within a dose range of 16.7–27.4 Gy. The heart DVH showed a maximum reduction of 2.35% within the dose range of 12.6–29.1 Gy. The LAD DVH showed the most significant reduction within the dose range of 15.1–37.0 Gy, with a maximum reduction of 15.76%. The DVH of the affected lung showed a maximum reduction of 0.87% within the dose range of 20.3–49.1 Gy. The results are shown in **Figure 4b**.

For laryngeal cancer, the mandibular DVH showed a maximum reduction of 2.03% within a dose range of 13.9–34.9 Gy. The oral cavity DVH showed a maximum reduction of 7.44% within a dose range of 29.2–39.9 Gy. The left parotid gland DVH showed a maximum reduction of 1.80% within the dose range of 34.9–56.1 Gy, while the right parotid gland DVH showed a maximum reduction of 1.62% within the dose range of 22.3–50.8 Gy. The pharyngeal constrictor DVH showed a maximum reduction of 5.23% within a dose range of 25.7–39.1 Gy. The spinal cord DVH showed a maximum reduction of 18.42% within a dose range of 13.8–22.4 Gy. The results are shown in **Figure 4c**.

### 3.5 Plan results

**Figure 5:**
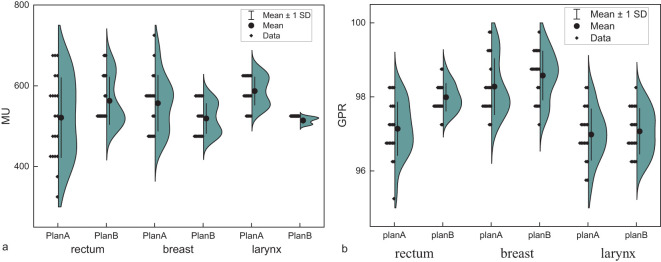
**(a)** Monitor units (MU) of Plan A and Plan B in rectal cancer, breast cancer, and laryngeal cancer. **(b)** The gamma pass rate (GPR) of Plan A and Plan B in rectal cancer, breast cancer, and laryngeal cancer.

The mean value of MU for rectal (p=0.022), breast (p<0.001), and laryngeal cancers (p<0.001) were 521, 557, and 587 for Plan A, and 562, 519, and 514 for Plan B, respectively. The results are shown in **Figure 5a**. The gamma pass rate (GPR) for all plans exceeded 95%. The results are seen in **Figure 5b**.

## 4 Discussion

This study compared VMAT plans using a 90˚ collimator angle (Plan B) with conventional collimator angle plan (Plan A) to evaluate its potential benefits in optimizing planning target dose distribution and protecting OARs. The results revealed the significant advantages of Plan B in key dosimetric indicators, providing innovative ideas for radiotherapy plan design and holding significant clinical application value.

The results indicated that Plan B was comparable to Plan A regarding target dose coverage, achieving 95% coverage of the prescription dose. This suggests no significant difference in the overall dose coverage capability between the two plans. However, Plan B was significantly superior to Plan A regarding GM and HI. The decrease in the GM of Plan B indicates a faster dose fall-off at the target margins, which could protect the surrounding normal tissues more effectively. Moreover, the reduction in the HI in Plan B suggests a more uniform dose distribution within the target. This may be attributed to the introduction of a 90˚ collimator angle, which fully uses the modulating capability of the MLC in the orthogonal direction, thereby improving dose distribution.

Regarding OAR protection, Plan B demonstrated a significant dose reduction trend compared with Plan A. In particular, for the intestine, which is located flexibly within the pelvic cavity and close to the target, Plan B significantly reduced the dose exposure to the intestine through the optimized collimator angles. This reduces the risk of complications caused by high-dose irradiation, such as enteritis and intestinal obstruction. Additionally, the decrease in the D_mean_ to the bladder indicates that Plan B can avoid increased dose exposure to the bladder, thereby reducing the incidence of bladder irritation symptoms. For breast cancer, Plan B reduces the dose to important organs such as the contralateral breast, heart, and left anterior descending artery (LAD). Although the V_5_ volume of the affected lung increased, the V_20_ decreased, which is favorable for protecting normal tissues overall. This optimization strategy helps reduce the long-term effect of radiotherapy on the heart and coronary arteries while minimizing damage to the affected lung. Notably, V_5_ and V_20_ for the left lung (LungL) exhibited considerable inter-patient variability, with standard deviations exceeding 20% of the mean in some cases. This variability may arise from differences in chest wall curvature, lung expansion, and beam entry geometry, all of which can affect low-dose bath distribution. As such, these parameters should be interpreted with caution, especially in small pilot cohorts. For laryngeal cancer, Plan B exhibited more precise dose control in areas such as the mandible, oral cavity, parotid glands, and pharyngeal constrictors, with a significant reduction in the maximum dose to the spinal cord. This indicates that Plan B offers greater advantage in protecting important organs and tissues in patients with laryngeal cancer, thereby effectively reducing the risk of radiotherapy-related complications.

In rectal cancers, the ratio of total body volumes receiving 20%, 40%, 60%, and 80% of the prescribed dose (V_20%_, V_40%_, V_60%_, and V_80%_, respectively) decreased in Plan B. This indicates that Plan B can better protect the surrounding normal tissues during rectal cancer radiotherapy while maintaining adequate tumor irradiation. However, for breast cancer, the changes in Plan B dose lines are more complex. The volumes receiving 20% of the prescribed dose increased, whereas those receiving 40%, 60% and 80% decreased significantly. These changes may be related to the specific shape of the breast cancer target and surrounding tissues. Overall, Plan B effectively controlled the high-dose region volumes in optimizing isodose lines, particularly in radiotherapy plans for rectal and breast cancers. This optimization strategy helps reduce the potential damage to the surrounding normal tissues while ensuring adequate planning target irradiation. For laryngeal cancer, optimizing dose lines in Plan B must consider the balance between the low- and high-dose regions to achieve the best therapeutic effect.

Through a comparative analysis of radiotherapy plans for the three types of cancer, we found significant differences in the mean number of MUs between Plan A and Plan B across different cancer types, with inconsistent trends. These differences may be closely related to factors such as tumor location, radiotherapy techniques, dose distribution optimization strategies, and individual patient anatomy. For example, Plan B may focus on protecting normal tissues within the pelvic cavity in optimizing rectal cancer. By increasing the number of MUs, a more complex dose distribution was achieved to reduce the dose exposure to normal tissues. In the breast cancer optimization process, Plan B may prioritize the uniformity and conformity of the dose distribution. Adjusting the arc angles and MLC movements reduces the demand for the MUs. For laryngeal cancer, Plan B may focus more on precise irradiation of the laryngeal tumor and the cervical lymphatic drainage area. A more precise dose distribution was achieved by reducing the number of MUs.

**Figure 6:**
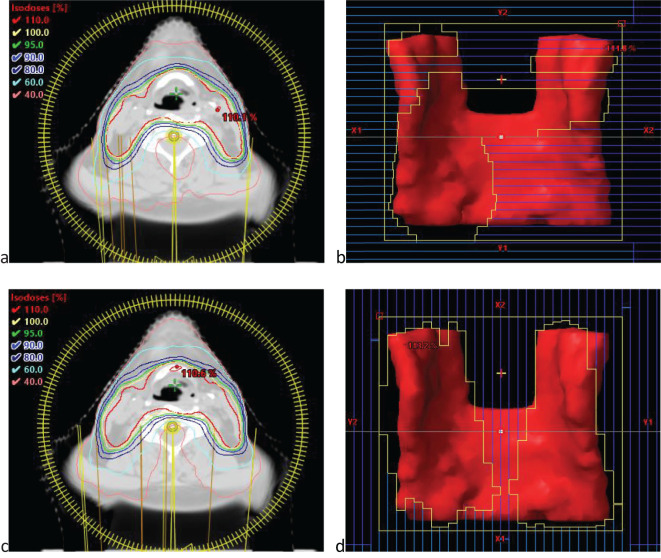
**(a)** The illustration of dose distribution under 0˚ collimator angles. **(b)** the beam eye view (BEV) under 0˚ collimator angles. **(c)** The illustration of dose distribution under 90˚ collimator angles. **(d)** the beam eye view (BEV) under 90˚ collimator angles.

Adjusting the collimator angle alters the propagation path and dose distribution of the radiation beam within a patient’s body. Introduction a 90˚ collimator angle allows the radiation beam to cover the target area more comprehensively, reducing the dose of cold and hot spots and achieving a more uniform and conformal dose distribution. Moreover, the complementary use of collimator angles at different angles can significantly reduce the dose exposure to OARs, particularly when the target has large curvature changes adjacent to or interwoven with OARs, and the advantages of the 90˚ collimator angle are more prominent. Target areas with large curvature changes have a concave shape, similar to the two separate targets. Such target structures are prone to problems similar to those of isolated island blockages when designed. **Figure 6** illustrates the dose distribution and beam eye view (BEV) for 0˚ and 90˚ collimator angles.

The HyperArc orthogonal collimator technique has garnered widespread attention owing to its ability to achieve high-precision dose irradiation among modern radiotherapy techniques. However, applying this technique requires complex equipment and optimization algorithms. By simplifying the technical approach, this study explores whether combining a 90˚ collimator arc with a 0˚ arc can achieve effects similar to those of orthogonal collimators while avoiding dose inhomogeneity issues. This study investigates the effects of applying the 90˚ collimator arc in radiotherapy and the rationality for its combination with the 0˚ arc. This approach achieves a high dose conformity similar to the Halcyon orthogonal collimator and effectively improves dose uniformity within the target. This combination optimizes the direction and intensity distribution of dose irradiation through the synergistic action of multiple angles, thereby reducing the dose inhomogeneity caused by a single arc angle.

Although personalized collimator angle settings can potentially achieve improved dosimetric results, they are often associated with increased planning complexity and require substantial time investment, experience, and technical expertise from physicists. In contrast, our study employed a fixed orthogonal configuration with collimator angles of 0˚ and 90˚ for the two arcs in Plan B, without additional modulation. This simplified approach not only enhances reproducibility and planning efficiency but is also feasible for implementation on most modern treatment planning systems and linear accelerators, which support assigning different collimator angles for each arc. Therefore, the proposed method offers a practical balance between dosimetric benefit and clinical applicability. They require extensive clinical experience and advanced planning and design skills. In comparison, adding a 90˚ collimator angle does not require complex adjustments for each case and has strong operability and universality. This can simplify the plan design process and improve work efficiency while ensuring therapeutic effects. In actual radiotherapy, when managing many patients with different types of tumors, there is a need for a method that can ensure therapeutic effects while simplifying the plan design process and improving work efficiency. The VMAT plan optimization method of adding a 90˚ collimator angle meets this demand and has significant clinical application value.

Despite the advantages of the method design and result analysis, this study has some limitations. First, the sample size was small, with only 60 patients, which may have limited the statistical analysis. This was a proof-of-concept investigation. Future research should validate the statistical effects and clinical applicability by expanding the sample size. Second, future studies should further explore the application effects of the 90˚ collimator angle at other tumor sites and its comprehensive application value in combination with other optimization techniques, such as adaptive radiotherapy. In addition, this study focused solely on improvement in dosimetric indicators and did not involve long-term follow-up of clinical treatment effects. Future research should combine patient clinical prognostic data to evaluate the efficacy and safety of the optimized plan for actual treatment.

## 5 Conclusion

In summary, by comparing the dosimetric indicators of conventional 0˚ collimator angle (Plan A) and 0˚ and 90˚ orthogonal collimator angles (Plan B) in VMAT plans, this study explored the effect of the 90˚ collimator on target dose distribution and OAR protection. The results demonstrated that Plan B significantly outperformed Plan A in key dosimetric metrics, including improved homogeneity index (HI) and gradient measure (GM), as well as reduced dose exposure to OARs, while maintaining complete target coverage and high-precision plan delivery. Moreover, the implementation of Plan B does not require complex individualized adjustments, offering high clinical feasibility and generalizability. This study provides a simple and effective new concept for designing radiotherapy plans with significant clinical value. Future research should expand the sample size to further validate its statistical effects and clinical applicability, and evaluate the actual efficacy and safety of the optimized plan.

## Data Availability

The raw data generated in this study contains clinical information that cannot be disclosed to the journal. Requests to access the datasets should be directed to XH, medicalphysicists@163.com.
